# A Neglected Case of Subacute Complete Uterine Inversion Managed by Haultain's Technique: A Rare Case

**DOI:** 10.7759/cureus.80296

**Published:** 2025-03-09

**Authors:** Poonam Lal, Swaroop R Nanda, Meena Samant

**Affiliations:** 1 Obstetrics and Gynecology, Kurji Holy Family Hospital, Patna, IND

**Keywords:** haultains procedure, peurperium, shock, subacute inversion, subtotal hysterectomy

## Abstract

Puerperal uterine inversion is a rare obstetric emergency and a potentially life-threatening condition of mismanaged third stage of labor. If not diagnosed immediately, the massive blood loss can lead to hypovolemic shock as well as neurogenic shock due to stretching of uterine nerve fibers and even maternal death. Diagnosis is mainly clinical. The diagnostic triad is hemorrhage, shock, and pain. Management includes manual uterine repositioning and correction of shock. However, in resistant or subacute cases that are rare, when a constriction ring is formed, incarceration occurs, and surgical intervention is needed to repose the uterus. We present a neglected case of rare subacute complete uterine inversion, which was managed by Haultain’s method followed by subtotal hysterectomy.

## Introduction

Puerperal uterine inversion is an uncommon yet life-threatening and unpredictable consequence of poorly managed third stage of labor. It is defined as the turning of the uterus inside out and can lead to severe hemorrhage, shock, and even maternal death [1]. The reported incidence is 1:2000 to 1:50,000 deliveries. It is more frequent in primigravida females and in those who delivered via cesarean section [2]. Maternal morbidity is significant in cases of uterine inversion, and the mortality rate could reach up to 15% [3].

Classification of uterine inversion according to an anatomic survey is as follows: first degree, fundus inverted below the level of the cervix; second degree, fundus inverted below the cervix but not up to the introitus; third degree, fundus inverted to the level of introitus; and fourth degree, complete uterine inversion with vaginal inversion [1].

Classification of uterine inversion according to delay between the delivery and diagnosis of uterine inversion is as follows [4]: acute inversion (83.4% cases), which occurs within 24 hours of delivery; subacute inversion (2.62% cases), which occurs between 24 hours and four weeks of delivery; and chronic uterine inversion (13.9% cases), which occurs after more than four weeks post-delivery. Diagnosis of uterine inversion is mainly clinical. There is a symptom triad of massive hemorrhage (70%), shock (30-40%), and pelvic pain (7-10%) [5].

Clinical signs are characteristic, with the absence of the uterine base, which is replaced by fundal cupping in first- and second-degree uterine inversions, whereas in complete inversion, there is an absence of the uterine fundus on per abdomen examination [2]. Inability to visualize or palpate the cervix on per vaginal examination and visualization of a fleshy and bloody mass that is exteriorized through the vulva are diagnostic of complete inversion [6]. The management is prompt recognition, manual uterine repositioning, and correction of shock. Manual uterine repositioning is possible before incarceration. If diagnosis is delayed, incarceration occurs due to constriction ring formation, and it becomes difficult to reposit the uterus manually [7]. Surgical intervention or correction such as Haultain’s technique may be needed in resistant and subacute uterine inversion [3].

We report a neglected case of subacute uterine inversion with failed attempts of manual repositioning managed by Haultain's technique and subtotal hysterectomy.

## Case presentation

A 23-year-old woman, P2 L1, presented on postpartum day 5 with complaints of a mass coming out per vaginum for two days. She delivered a full-term female baby weighing 2.5 kg vaginally at a private nursing home. She had an episode of postpartum hemorrhage for which she received 3 units of blood. After 72 hours of delivery, she noticed a huge mass coming through the vagina on straining during defecation. Repeated attempts were made in a hospital to repose it under anesthesia but failed, after which she was referred to our hospital on postpartum day 5. She was conscious and well-orientated to time, place, and person. Her pulse rate was 98 beats/min with good volume, blood pressure was 100/70 mm Hg, and temperature was 100 degrees F. Her hemoglobin level was 9.6 g/dL, total leukocyte count was 20,000/mm³, and the rest of the investigations such as liver function test, kidney function test, and viral markers (HIV, HBSAg, HCV), were normal.

**Figure 1:**
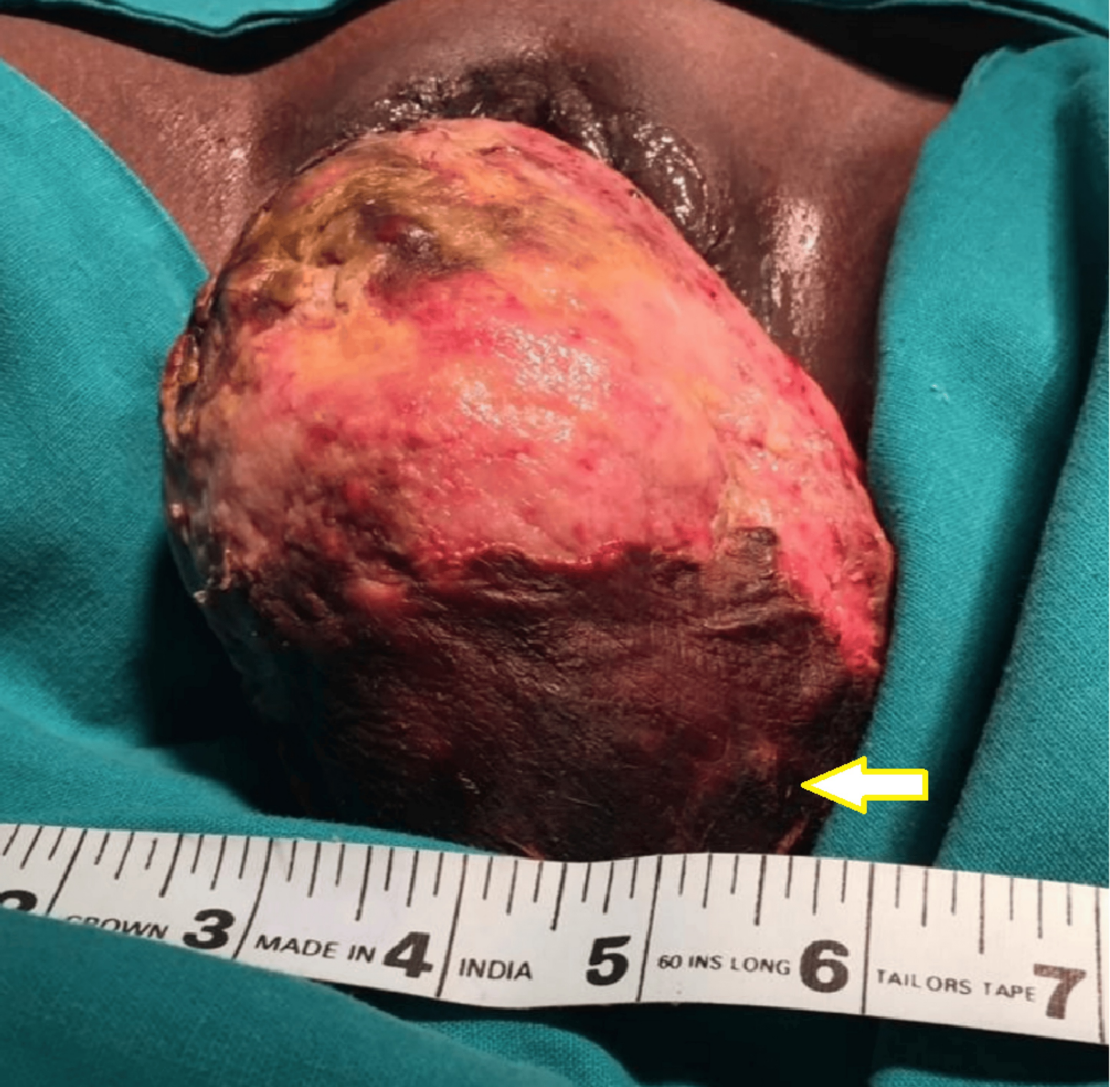
Huge fleshy mass seen on inspection The yellow arrow points to the infected surface of uterine fundus

On local examination, a large, soft, infected, red-colored mass with areas of blackish discoloration was seen coming through the vulva on inspection (Figure 1). On per abdominal examination, we could not find the uterine fundus. The cervix could not be felt separately on per vaginal examination. On transabdominal USG, the uterine fundus could not be appreciated, and a diagnosis of uterine inversion was made. The complete diagnosis of a neglected subacute complete uterine inversion was made.

**Figure 2:**
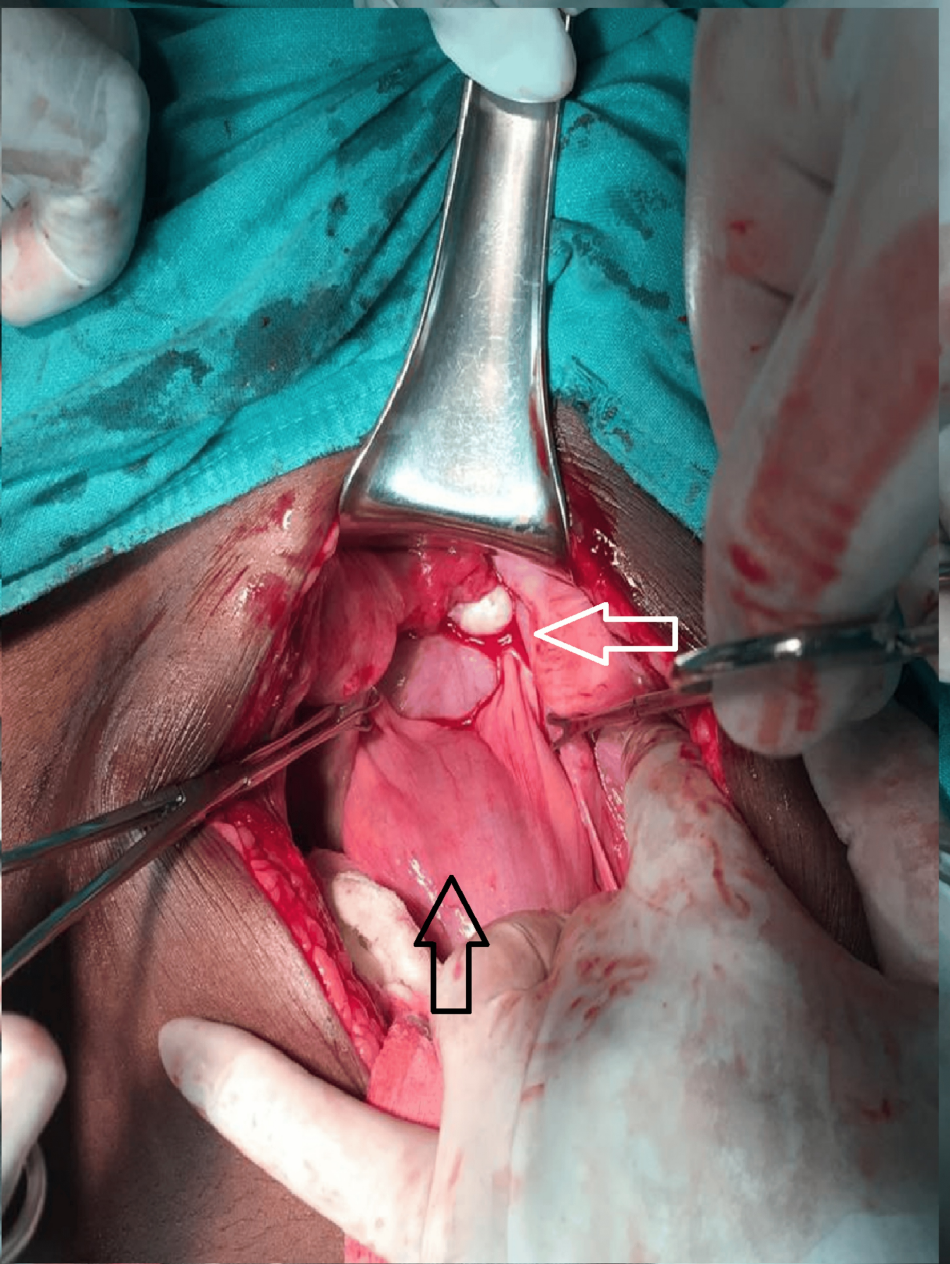
Intraoperative finding illustrating an absent uterine fundus The black arrow points to the bladder, and the white arrow points to the absence of uterine fundus with pulled-in adnexal structures

**Figure 3:**
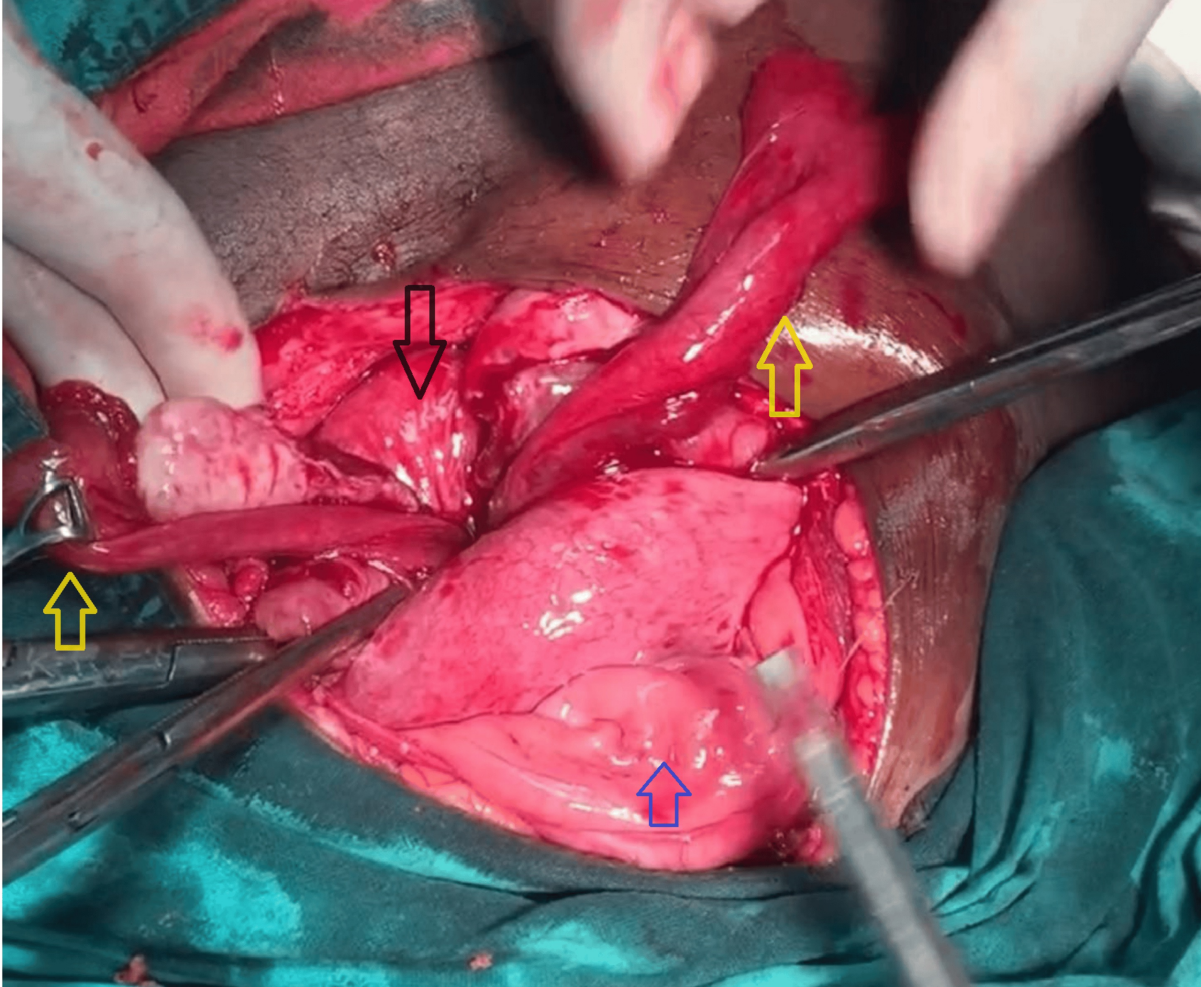
Flower vase appearance in uterine inversion with constriction ring The blue arrow points to the bladder surface, the black arrow points to the posterior part of the constriction ring, and the two yellow arrows point to the adnexal structures pulled inside the constriction ring

**Figure 4:**
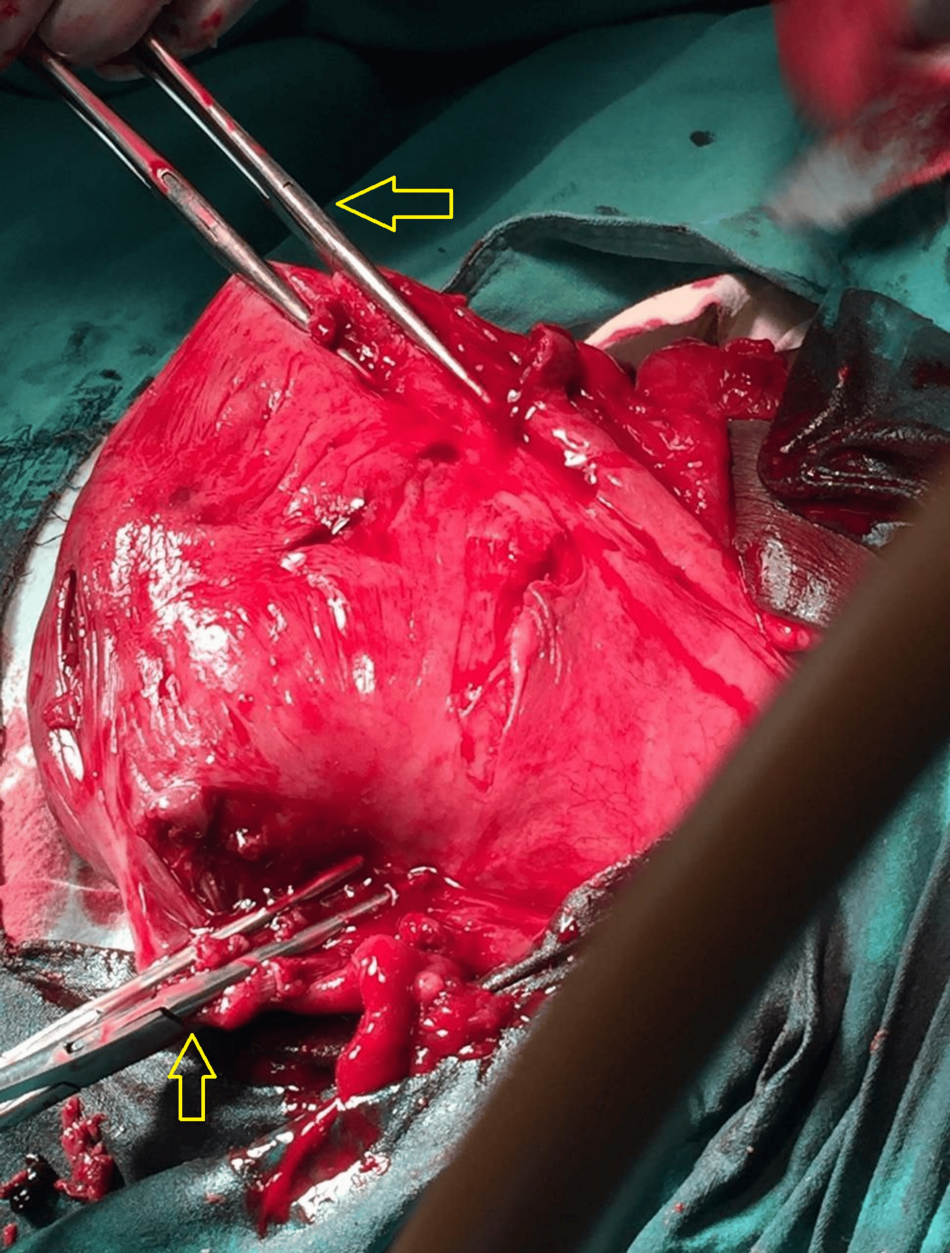
Intraoperative picture demonstrating a successfully reposited uterus The yellow arrows show the Allis forceps used for traction on the round ligaments

Blood culture reports isolated *Escherichia coli* sensitive to piperacillin. We started her on a combination of piperacillin and tazobactam with clindamycin. After arrangements of blood and blood products, we transfused one unit of packed red blood cells preoperatively. Taking informed consent for hysterectomy, we proceeded with laparotomy. The abdomen was opened in layers, and we could not find the uterine fundus, as illustrated in Figure 2. A constriction ring was identified with difficulty after making traction on the bilateral round ligament with Allis forceps and with simultaneous pushing up of the uterus vaginally. The constriction ring can be seen in Figure 3. An incision was made posteriorly to release the constriction ring. The uterus was reposed within the abdominal cavity by a fist passed through the vagina. Figure 4 illustrates the successfully reposited uterus. There were no bladder and rectal injuries observed during the procedure.

**Figure 5:**
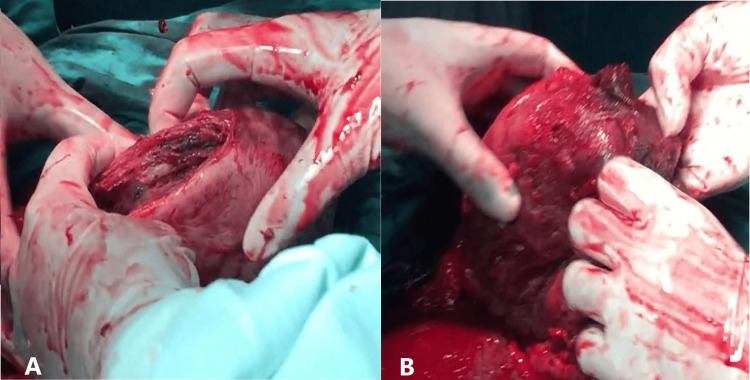
Intraoperative picture showing infected uterine surface (A) The torn left wall of the uterus. (B) The infected endometrial surface.

A subtotal hysterectomy was performed as the endometrial surface was infected, as shown in Figure 5B. There was an intraoperative blood loss of 450 mL. Her postoperative hemoglobin level was 9 g/dL, and the rest of the postoperative period was uneventful. She was discharged on post operative day 5.

## Discussion

Uterine inversion is defined as the uterine fundus turning inside out through the endometrial cavity and cervix [8]. The exact cause of uterine inversion is still unknown and unexplained. Extrinsic risk factors include oxytocic arrest after a prolonged labor, umbilical cord traction, and abdominal pressure [9]. Instrinsic factors are also reported, such as primiparity and rapid emptying of the uterus after prolonged distension [10]. Other intrinsic factors are uterine hypotonia secondary to twin pregnancy, placenta accreta, and short umbilical cord [5].

Puerperal uterine inversion is usually a clinical diagnosis. The triad of elements is hemorrhage, shock, and strong abdominopelvic pain [2,11]. In more than 70% of cases, the bleeding is substantial, and the shock is the most persistent sign, which arises from hypovolemia due to massive bleeding and neurogenic shock from vagal stimulation due to stretching of nerve fibers contained in the uterine segment [12,13]. Around 7-10% cases of uterine inversion present with severe pain of sudden onset [6].

Acute uterine inversion requires prompt repositioning under antimicrobial coverage. Uterine repositioning can be accomplished non-surgically by manual repositioning of the uterine fundus or the hydrostatic approach known as the O'Sullivan method [14].

If non-surgical uterine repositioning fails, surgical repositioning or hysterectomy may be necessary. Surgical intervention is usually necessary in chronic uterine inversions that are difficult to repose manually owing to the reduced elasticity of uterine walls [15]. An abdominal operation and vaginal operation are both viable options for treating subacute and chronic inversion. Two abdominal surgeries are Haultain's technique (the cervical ring is incised posteriorly to facilitate uterine replacement) and Huntington's method (the cervical ring is incised anteriorly) [16], and two vaginal surgeries are Spinelli’s and Kustner’s techniques, which involve replacing the uterine fundus through anterior and posterior transection, respectively [16].

Compared to the vaginal route, the abdominal route is the preferred option due to the reduced uterine incision, simple repositioning due to traction on the round and broad ligaments, and easy approximation and accurate suturing of the uterine wall [15].

Watson et al. reported 18 cases of acute and subacute puerperal inversion. The most common signs noted were hemorrhage (94%) and shock (39%). All inversions were recognized immediately and manually replaced within 60 minutes. Calculated blood loss was 1,775 mL. There was no mortality or febrile morbidity. The average hospital stay was three days [5].

Sinha et al. [17], Ali and Kumar [18], and Sharma et al. [3] managed a neglected case of subacute inversion by Haultain’s repair. A case report by Effendy et al. [19] documented successful repositioning of the uterus in the case of subacute uterine inversion with the help of Haultain's technique and could save the uterus. A case of subacute inversion in a primigravida was also reported by Chandrayan and Dobariya [20], which was managed by Haultain's technique followed by uterine preservation.

Our case was neglected as the patient presented to us on postpartum day 5, when sepsis had already set in, and intraoperatively we found an infected endometrial surface suggestive of possible endomyometritis. Therefore, we proceeded with subtotal hysterectomy with consent to save her life.

## Conclusions

Since uterine inversion is rare, there is a lack of experience in tackling this obstetric emergency. The incidence and complications of uterine inversion can be substantially reduced through early recognition and active management of third stage of labor. Certain maneuvers to be avoided are extra traction on the umbilical cord, excessive fundal pressure, excessive intra-abdominal pressure, and excessively vigorous manual removal of the placenta. Manual uterine repositioning is often successful; however, in resistant or subacute cases, surgical correction might be needed. Therefore, obstetricians must update themselves about the techniques to solve this emergency for good maternal outcomes.
